# Renal Endothelial Cytotoxicity Assay to Diagnose and Monitor Renal Transplant Recipients for Anti-Endothelial Antibodies

**DOI:** 10.3389/fimmu.2022.845187

**Published:** 2022-06-06

**Authors:** Rosa G. M. Lammerts, Jacob van den Born, Magdalena Huberts-Kregel, Antonio W. Gomes-Neto, Mohammed R. Daha, Bouke G. Hepkema, Jan-Stephan Sanders, Robert A. Pol, Arjan Diepstra, Stefan P. Berger

**Affiliations:** ^1^Department of Nephrology, University Medical Center Groningen, University of Groningen, Groningen, Netherlands; ^2^Transplantation Immunology, Department of Laboratory Medicine, University Medical Center Groningen, University of Groningen, Groningen, Netherlands; ^3^Department of Surgery, University Medical Center Groningen, University of Groningen, Groningen, Netherlands; ^4^Department of Pathology and Medical Biology, University of Groningen, University Medical Center Groningen, Groningen, Netherlands

**Keywords:** complement biology, crossmatch, cytotoxicity, desensitization, flow cytometry, non-HLA

## Abstract

Tissue-specific nonhuman leukocyte antigen (HLA) antigens can play crucial roles in allograft immunity and have been shown to trigger humoral responses leading to rejection of HLA-matched kidney allografts. Interest in the role of endothelial-specific antigens has grown over the past years, and several case reports have been described in which antibodies reacting with endothelial cells (ECs) are associated with rejection. Such antibodies escape the detection in conventional crossmatch tests as they do not react with lymphocytes. However, due to the heterogeneity of endothelial cells from different vascular beds, it remains difficult to draw organ-specific conclusions from studies describing endothelial crossmatch assays. We present a case of a 69-year-old male patient whose kidney allograft was rejected as hyperacute, despite the absence of pretransplant HLA-specific antibodies. To place findings from previous studies in a kidney-related context, we performed crossmatch assays with primary renal endothelial cells. The patient’s serum was reactive with primary renal ECs, demonstrated by antibody binding and complement-dependent cytotoxicity. Antibodies from this patient did not react with lymphocytes nor were HLA donor-specific antibodies (DSAs) found. Two years later, the patient successfully received a second kidney transplant after treatment with rituximab and plasmapheresis before and after transplantation. We demonstrated that the removal of antibodies against non-HLA EC-specific molecules can be monitored using a primary renal EC crossmatch test, possibly contributing to a successful transplantation outcome.

## Introduction

The presence of donor-specific human leukocyte antigen (HLA) antibodies in patients awaiting a renal transplant can either be a contraindication for transplantation or pose an increased risk for antibody-mediated rejection (ABMR) and inferior graft survival (1). However, unexpected ABMR episodes still occur despite thorough pretransplant screening with the current routinely used techniques. This might be explained by HLA-specific memory cells that become activated upon re-exposure to the antigen or the emergence of *de novo* antibodies after transplantation. Non-HLA antibodies are also related to graft loss in the absence of HLA antibodies and are generally not detected by routine crossmatching with lymphocytes. In the past years, a number of non-HLA antigens have been identified in kidney transplantation, including angiotensin type 1 receptor, endothelin type A receptor, collagen-V, K-α1 tubulin, and perlecan (2, 3). Non-HLA antiendothelial antibody (AECA) concentrations are higher in sera from kidney transplant recipients with acute or chronic rejection compared to stable transplant recipients (4, 5). Screening for non-HLA AECA has not yet been implemented in clinical practice, despite increasing evidence for these antibodies to be involved in rejection. Non-HLA AECA could be detected by endothelial cell (EC) crossmatches, and the appearance of AECA was associated with an increased risk of allograft rejection (6–8). However, these studies all have the disadvantage of not utilizing renal EC and therefore underestimating the tremendous heterogeneity of ECs derived from different origins in the human body (6, 8–14). Recently, Crespo et al. described that a positive endothelial cell crossmatch (ECXM) using aortic endothelial cells did not correlate with the histology of ABMR, neither using pretransplant serum nor posttransplant serum. Also, positivity in the EXCM results was found in all investigated patient groups: in patients with normal renal histology, histology of interstitial fibrosis, and tubular atrophy and histology of ABMR. This positivity was not associated with any histological signs of endothelial damage, e.g., ABMR histology. However, as the authors also clearly stated themselves, the EC crossmatch was performed with aortic ECs, which may not express the same proteins as renal ECs (15). Delville et al. showed the relevance of a non-HLA AECA crossmatch test using a renal glomerular endothelial cell line (CiGeNC), confirming specific renal microvascular EC responses and revealing substantial differences in transcriptomic profiles between macrovascular and microvascular ECs (7).

Crossmatching using one cell line does not address the variability of expressed antigens between individuals, which may form the basis for non-HLA antibody formation. In addition, the question of how to treat and monitor patients with confirmed anti-non-HLA AECA remains unanswered to date (16). Strategies that are established in the event of HLA antibody-mediated rejection and blood group-incompatible transplantation are based on rapid and effective reduction of antibody titers. We hypothesized that this could also be applicable in the area of non-HLA antibody mediated rejection (17).

This report presents the case of a patient who developed hyperacute allograft rejection in the absence of HLA-specific antibodies, both before and after rejection occurred. We demonstrate the value of EC based cross matching (18), and describe the patient’s successful re-transplantation, monitored with our EC-based crossmatch assay.

## Materials and Methods

### Human-Leukocyte Antigen Typing and Human-Leukocyte Antibody Detection

The HLA typing of a male patient, aged 67, diagnosed with rapidly progressive glomerulonephritis and his donor was performed with sequence-specific oligonucleotide primer (SSOP) technology, analyzed with the IMGT/HLA allele database 3.23. The detailed description of the case is depicted in the section describing the clinical history.

**Figure 1:**
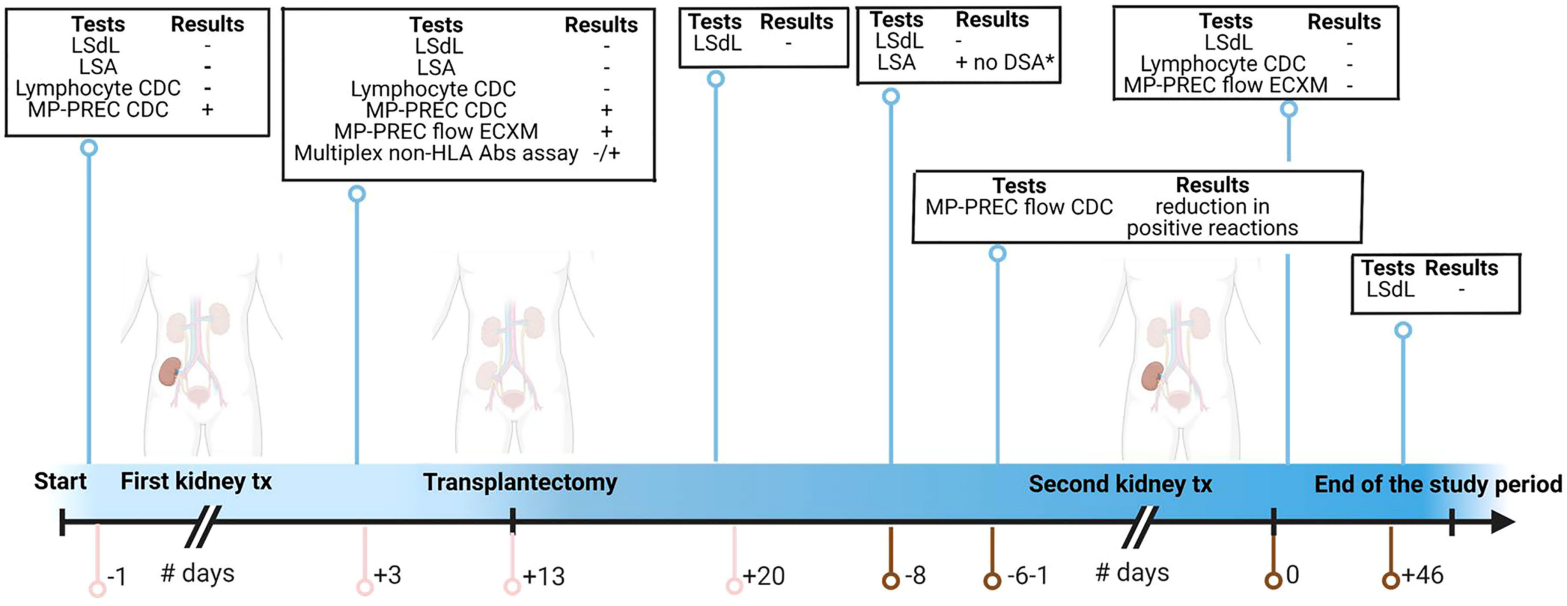
Timepoints for samples obtained and tests performed. The pink line represents the days related to the first kidney transplantation; the brown line represents the days related to the second kidney transplantation. tx, transplantation; LSdL, Life Screen Deluxe; LSA, Luminex single-antigen bead assay; CDC, complement-dependent cytotoxicity; MP-PREC, machine perfusion derived primary renal endothelial cells; ECXM, endothelial cell crossmatch; DSA, donor-specific antibodies Figure created with BioRender.com.

The presence of HLA class I and II Abs in the patients’ serum was evaluated using the Life Screen Deluxe (LsdL), in accordance with the manufacturer’s protocol (Immucor GTI Diagnostics Inc., Waukesha, United States, lot. 3003946-3003920)) and with the Lifecodes Single Antigen Bead (LSA; Immucor Transplant Diagnostics) assay. The routinely lymphocyte-based complement-dependent cytotoxicity tests were used to test for panel-reactive and donor-specific antibodies. An overview of HLA antibody detection and endothelial cell crossmatch (ECXM) tests and timepoints is given in **Figure 1**.

### Staining of CD34, ERG, IgG, IgM, C4d, and C3d in Renal Biopsies

Pre- and postimplantation biopsies were stained with endothelial-specific marker mouse anti-human CD34 (QBEnd/10,Ventana, Mannheim, Germany) and rabbit anti-human ETS-related gene (ERG) (ERP3864, Ventana, Mannheim, Germany) according to the manufacturer’s protocol, as part of the standardized pathological evaluation of biopsies. Postimplantation biopsies were stained with goat anti-human IgG (760-2680, Ventana, Benchmark automated immunostainer), goat anti-human IgM (760-2682, Ventana, Benchmark automated immunostainer), and rabbit anti-human C4d (clone SP91, Ventana, Benchmark automated immunostainer) also according to the manufacturer’s protocol, as part of the standardized pathological evaluation of biopsies. C3d staining was performed using in-house staining. Solutions were prepared using with BSA (A9647, Sigma, St Louis, United States) and PBS (17-512Q, Lonza, Wijchen, The Netherlands) In short, deparafffinization was performed using xylol and alcohol (100%, 96%, 70%) and demi-water in 6 consecutive steps. This was followed by incubation in the dark for 30 min, at 37°C, with 0,4% pepsin from porcine stomach mucosa in 37% 0.1N HCl in demi-water (pH = 2.5). A blocking step was then performed by first adding 0.01% H_2_O_2_ (in 1% BSA with PBS) followed by 1% BSA in PBS. The polyclonal rabbit antihuman C3d (A0063, Dako, Glostrup, Denmark) at 4°C overnight in a 1:2,500 dilution in 1% BSA in PBS was then added. This was followed by the first conjugate, HRP polyclonal goat anti-rabbit antibody (P0448, Dako), at room temperature in a 1:100 dilution in 1% BSA in PBS and the second conjugate HRP on polyclonal rabbit anti-goat antibody (P0449, Dako) at room temperature in a 1:100 dilution in 1% BSA in PBS. The substrate was then added with 0.2 mg/ml 3-amino9-ethylcarbazole (02431MH, Sigma), in 50 mM acetate buffer and 0.03% H_2_O_2_ (pH = 5.5) followed by a counterstaining and embedment using hematoxylin 1:2 for 5 s and a Kaiser’s glycerol gelatine embedment (1092420100, Merck, Darmstadt, Germany). An overview of all antibodies used is given in **Supplementary Table S1**.

### Endothelial Cell-Based Complement-Dependent Cytotoxicity Test

To provide direct evidence for the presence of potentially pathologic (i.e., cytotoxic) alloantibodies against the machine perfusion-derived primary renal endothelial cells (MP-PRECs), a cytototic crossmatch was performed combining patient serum and primary renal endothelial cells (ECs). If non-HLA or HLA antibodies are present in patient serum, these antibodies can bind the ECs and form antibody–antigen complexes that activate the complement cascade, leading to complement-mediated cytotoxicity. The Terasaki typing trays were prepared as follows: 10 µl patient sera was added to the Terasaki wells. As positive control serum containing HLA antibodies directed against HLA-A2 (A2/A28 IgG1 and IgM, provided by Dr. F. Claas, Leiden University Medical Center, The Netherlands) expressed on the endothelial cell was used. As negative control normal human serum (NHS) from healthy volunteers with a compatible blood group and without HLA antibodies was used. Culture MP-PRECs were dissociated using nonenzymatic cell dissociation solution (C5789, Sigma^®^, Zwijndrecht, The Netherlands). Cells were isolated and characterized as described before, and clinical baseline characteristics regarding the donors of the MP-PRECs can be found in our recently published paper describing the isolation and characterization of the MP-PRECs in detail (18). Experiments were performed with cells cultured up to passage 3–5. The cells were pelleted and resuspended with culture medium (Medium 200 (GIBCO, Cat#M-200-500, Grand Island, NY, United States) containing Low Serum Growth Supplement (LSGS, GIBCO, Cat#S-003-K, Grand Island, NY, United States) for a final concentration of 2000cells/µl. 1 µl of the cell suspension was added to each terasaki well. Cells were incubated for 30 minutes with sera followed by 60 minutes 5 µl rabbit complement or 60 minutes with sera followed by 120 minutes 5 µl rabbit complement at room temperature. Ethidiumbromide and acridineorange were added to stain for apoptotic and living cells. The reaction was stopped by adding sodium detaat. Results are expressed as percentage of apoptotic cells in relation to the total amount of cells.

A confocal inverted laser microscope (Leica) was used for acquisition of the staining. The fields, systematically digitized throughout the well, were acquired using a computer-based image analysis system. The staining was quantified using built-in specific functions of the software Image J (NIH, Bethesda, MD) and expressed as percentage per well.

### Flow Cytometry

To explore classical pathway complement activation, MP-PRECs were cultured in a 12-well culture plate until they reached confluence and were detached using cell dissociation solution (C5789, Sigma^®^, Zwijndrecht, The Netherlands), 900 μl/1 ml at 37°C, collected in 4.5 ml tubes containing 2 ml cell of medium and centrifuged twice at 250×*g* for 6 min at 20°C. Thereafter, MP-PRECs were incubated with 25% blood group-compatible heat-inactivated NHS, or 25% blood group-compatible heat-inactivated serum containing HLA-A2 directed against the antigens on the cell membrane, or 25% of the patient’s serum, diluted in M200 medium (M200 culture medium, Ref. No. M200500, Gibco, Bleiswijk, The Netherlands) supplemented with low growth serum (supplement kit, Ref. No. S-003-K, Gibco, Bleiswijk, The Netherlands) for 45 min at 37°C. Hereafter, cells were centrifuged twice for 6 min at 250×*g* at 20°C in culture medium, followed by incubation with 20% NHS as a complement source for 30 min at 37°C. After incubation, MP-PRECs were washed twice with ice-cold phosphate-buffered saline (PBS)/1% bovine serum albumin (BSA) (FACS buffer) (Sigma^®^, Zwijndrecht, The Netherlands) at 250×*g* for 6 min at 4°C. For detection of IgG(G18-145, BD Biosciences, San Jose, United States), IgM (MHM-88, BioLegend, San Diego, CA, United States) and complement activation [activated C3 (HM2168, Hycult, Uden, The Netherlands)], the MP-PRECs were incubated with antibodies depicted in **Supplementary Table S1**, for 30 min on ice. Cells were washed twice with ice-cold FACS buffer, centrifuged at 250×*g* for 6 min at 4°C, and incubated with goat anti-mouse FITC (Southern Biotech, Birmingham, United States) for 30 min on ice in the dark. Propidium iodide at 1 μg/ml (Molecular Probes, Leiden, The Netherlands) was added just before measuring to be able to exclude apoptotic and necrotic cells. Activated C3 deposition on viable nonapoptotic cells were analyzed in a FACSCalibur™ (FACSCalibur, Becton Dickinson, New Jersey, United States). The results are from at least three independent experiments. The percentage effect was calculated based on the control data (blood group and HLA-compatible serum) in mean fluorescence intensity (MFI); % binding is = (MFI test result/MFI control) × 100.

### Complement Source and Handling

Rabbit complement (ThermoFisher Scientific CL3115, Cedarlane, Burlington, Canada) and normal human serum (NHS) from healthy volunteers having blood group AB were used separately as a complement source. NHS and rabbit complement were stored at −80°, rapidly thawed at 37°C and diluted in M200 medium to a final concentration of 25% for human complement and 20% for rabbit complement prior to use. As control, NHS was incubated at 56°C for 30 min to inactivate complement proteases.

### Multiplex Assay

The patient’s serum from postoperative day 3 was measured using the multiplex assay. The development, technical details, and validation of this high-throughput multiplex assay for the detection of non-HLA antibodies are described extensively in the study by Kamburova et al. (19)

### Statistical Analysis

Statistical tests were conducted for complement component measurements. Differences in the percentage effect and percentage apoptosis were analyzed by the unpaired *t*-test. Computations were performed by SPSS (IBM Statistics Chicago, United States) version 23, and GraphPad software 8.0 (Graphpad Software, San Diego, United States) was used for graphical visualization.

## Results

### Clinical History

The patient presented in July 2015, aged 67, with rapidly progressive glomerulonephritis, a serum creatinine of 583 µmol/l and an anti-glomerular basement membrane (anti-GBM) antibody titer of >600 IU/ml without pulmonary involvement. He was diagnosed with anti-GBM glomerulonephritis based on the clinical parameters and a positive anti-GBM titer but not by kidney biopsy. He was treated with hemodialysis, plasmapheresis, cyclophosphamide, and prednisone without improvement of kidney function. During this treatment, he received one blood transfusion. The patient’s 70-year-old brother was considered eligible for kidney donation, and in November 2016, the patient received a living-related AB0 compatible kidney transplantation. Donor and recipient HLA typing was A2, A68, B62, B44, Bw6, Bw4, Cw1, Cw2, DR4, DR14, DR52, DR53, DQ8, and DQ5 and A2, A68, B7, B44, Bw6, Bw4, Cw2, Cw7, DR14, DR15, DR52, DR51, DQ5, and DQ6 for donor and recipient, respectively, resulting in a 0-1-1 HLA mismatch. The donor and recipient both tested positive for the Epstein–Barr and cytomegalo viruses. The last anti-GBM titer before transplantation was 14 IU/ml. No panel reactive and donor-specific antibodies were detected prior to transplantation in the lymphocyte based CDC test, LSdL, and LSA assay.

### First Post-transplantation Clinical Course

**Figure 2:**
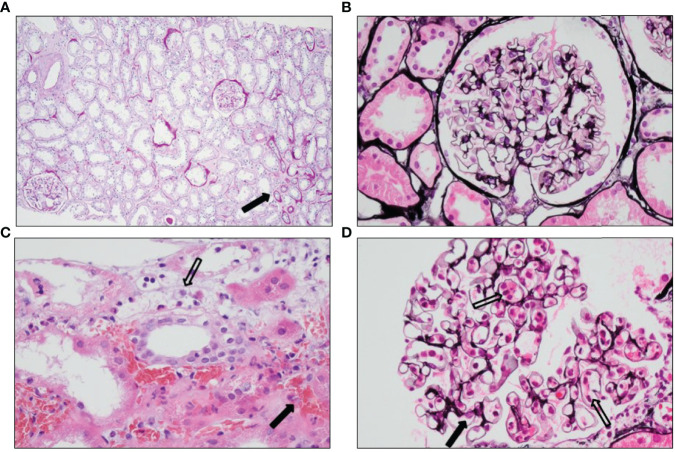
Histology of the pre- and post-implantation biopsies. **(A)** PAS staining showing no pre-existent damage apart from <5% atrophy (arrow) seen as broadened tubular basement membranes, fibrosis, and smaller tubuli in the preimplantation biopsy. **(B)** Jones’ methenamine silver staining showing a normal glomerulus in the preimplantation biopsy. **(C)** Hematoxylin and eosin staining showing hemorrhagic areas outside the vascular bed (black arrow) and inflammatory cells with destruction of endothelial cells (open arrow) in the posttransplantation biopsy. **(D)** Jones’ methenamine silver staining showing slightly expanded glomerular capillaries with abundant inflammatory cells (open arrows) and somewhat pronounced endothelial cells (black arrow) in the posttransplantation biopsy.

No relevant pre-existing damage was found in the routine preimplantation biopsy of the kidney (**Figures 2A, B**), and no surgical complications occurred (cold ischemia time, 142 min; warm ischemia time, 33 min) and renal perfusion, as determined by Doppler ultrasound, was excellent with immediate diuresis. Serum creatinine values declined from 940 to 514 µmol/L after transplantation. The immunosuppressive regimen consisted of mycophenolate mofetil (1,000 mg bd), tacrolimus (0.075 mg/kg bd), and prednisone (40 mg iv qd), according to the standard protocol. Within hours after transplantation, the patient developed a fever and diuresis dropped from 350 to 75 ml/h.

On postoperative day (POD) 1, nasopharyngeal, urine, and blood cultures were performed, and prophylactic ceftriaxone was started. Diuresis declined to 25 ml/h without a response to fluid challenges and cultures returned negative.

**Figure 3:**
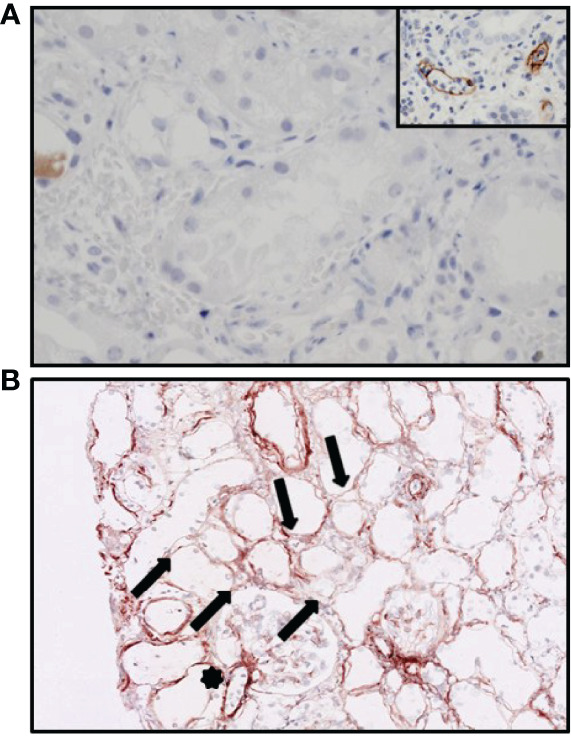
Immunohistochemistry of the hyperacutely rejected allograft. **(A)** Staining for C4d showing no presence of C4d in the peritubular capillaries. The inset shows a positive control with acute humoral rejection. **(B)** C3d staining showing variable staining along the peritubular capillaries (arrows) and hilus of the glomerulus (star).

**Figure 4:**
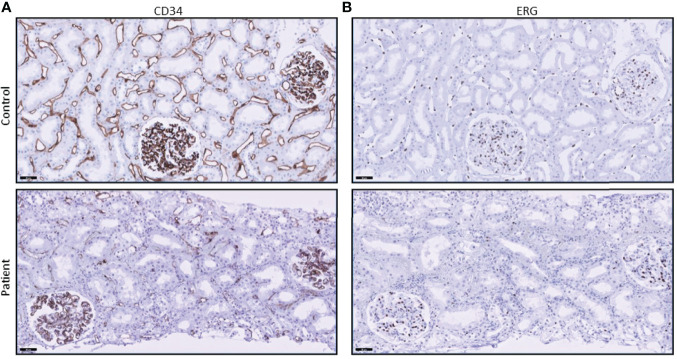
CD34 and ERG stainings show loss of endothelial cells. **(A)** CD34 staining in the peritubular capillaries of the patient’s biopsy is diminished when compared to control tissue (posttransplant biopsy without signs of rejection). **(B)** ERG staining also shows a loss of endothelial cell nuclei in the peritubular capillaries.

On POD 2, diuresis was 430 ml/24 h accompanied with hematuria. Doppler ultrasound showed diminished perfusion of the kidney with moderate swelling of the renal parenchyma and no signs of renal vein or artery thrombosis. Thrombocytes declined from 117 × 10 (9/L to 59 × 10^9^/L, C-reactive protein values increased to 197 mg/L, serum creatinine values increased to 650 µmol/L, and anti-GBM titer was 10 IU/ml. A Tc-99m MAG3 scintigram revealed poor perfusion of the transplanted kidney; however, during re-exploration surgery, no vascular complications were found, though the kidney had a purple discoloration and was swollen and stiff. A biopsy taken during re-exploration revealed granulocytes and lymphocytes in the glomerular capillary loops with occasional small amounts of intraluminal fibrin. Granulocytes were also present in the peritubular capillaries and associated with detachment and destruction of endothelial cells. There was extensive peritubular hemorrhage (**Figures 2C, D**). The tubuli appeared ischemic, with flattening of the epithelial cells. No arterial necrosis was found. Immunoglobulin G (IgG) and immunoglobulin M (IgM) staining by immunofluorescence was barely seen (**data not shown**), and the C4d staining was negative (**Figure 3A**). Additional staining for endothelial cell-specific markers CD34 and ERG showed less CD34- and ERG-positive cells compared to a posttransplantation biopsy without rejection (**Figures 4A,B**). This was most apparent in the peritubular capillaries, indicating a pronounced loss of endothelial cells. C3d staining was most pronounced in the peritubular capillaries and the glomerular hilus (**Figure 3B**). Despite the negative C4d staining, humoral rejection was strongly suspected; hence, prednisone, intravenous immunoglobulin (IVIg), and plasmapheresis was started. On POD 3, no HLA antibodies were found in the LSdL and the LSA assay, nor against A/B/DR loci, nor against C, DQ, and DP loci. In addition, serum creatinine increased to 740 µmol/L, and the patient required dialysis again. During the following days, the patient continued to be treated with plasmapheresis, IVIg, and prednisone. Despite this therapy, the kidney function did not improve, and this resulted in a transplantectomy on POD 13.

### MP-PREC Non-HLA Tests

Despite the pathological signs of hyperacute ABMR with capillaritis, we could not detect HLA antibodies in patient serum obtained pretransplant or on POD3. Therefore, non-HLA-mediated rejection was considered. To determine this, we crossmatched the patient’s serum taken 1 day prior to transplantation and from POD 3 with human MP-PRECs, utilizing rabbit complement.

**Figure 5:**
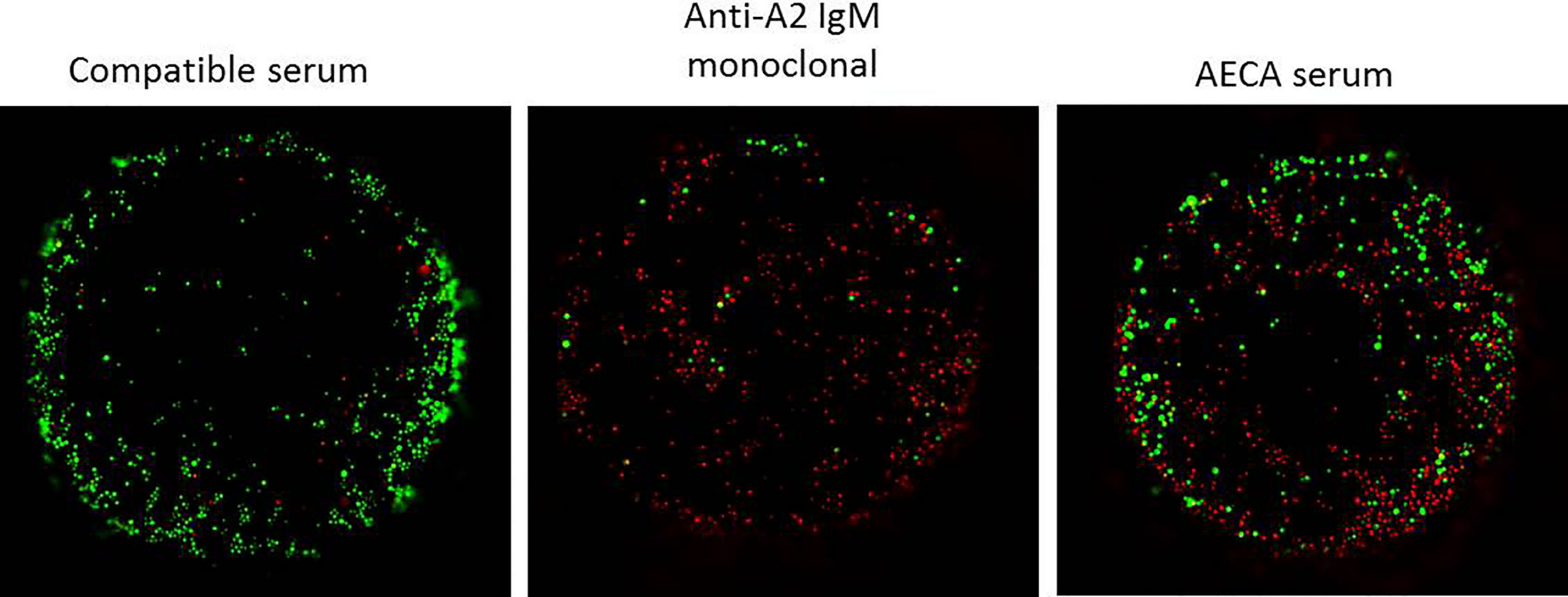
Complement-dependent endothelial cytotoxicity using machine perfusion-derived primary renal endothelial cells (MP-PRECs) and pre-transplant patient serum. A representative experiment of a complement-dependent endothelial cytotoxicity crossmatch assay (CDC) using MP-PRECs (HLA typing A1, A2, B7, B8, Bw6, Cw7, DR15, DR11, DR51, DR52, DQ6, DQ7, and bloodgroup A, incubated with blood group-compatible serum, a monoclonal antibody directed against A2 IgM, and the AECA-positive patient serum. Rabbit complement was used as the complement source. Living cells are depicted in green and apoptotic cells in red.

**Figure 6:**
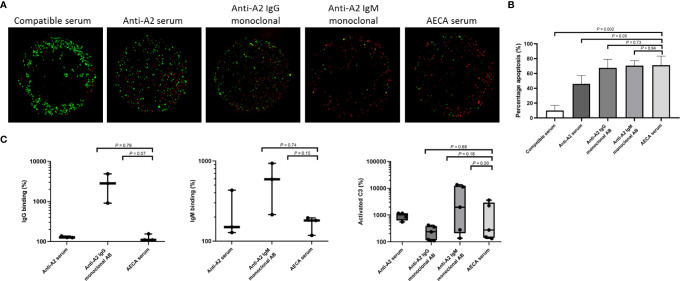
Complement-dependent endothelial cytotoxicity and flow crossmatch assays using machine perfusion-derived primary renal endothelial cells (MP-PRECs) and post-transplant patient serum. **(A)** A representative experiment of a complement-dependent endothelial cytotoxicity crossmatch assay (CDC) using MP-PRECs (HLA typing A2, B62, B60, Bw6, Bw6, Cw10, DR1, DR4, DR53, DQ7, DQ5, and bloodgroup A, incubated with blood group-compatible serum, blood group- and HLA-incompatible serum (anti-A2 serum), a monoclonal antibody directed against A2 IgG, a monoclonal antibody directed against A2 IgM, and the AECA-positive patient serum. Rabbit complement was used as the complement source. Living cells are depicted in green and apoptotic cells in red. **(B)** Quantification of *n* = 3 experiments of apoptotic (red) cells. *p-*values are depicted in the figure. **(C)** Flow cytometry showing IgG, IgM, and activated C3 binding in percentages after incubation of MP-PRECs with blood group- and HLA-compatible serum, blood group- and HLA-incompatible serum (anti-A2 serum), a monoclonal antibody directed against A2 IgG, a monoclonal antibody directed against A2 IgM, and the patients’ serum having AECA. A total of 20% human serum was used as the complement source. MP-PRECs that were propidium iodide negative were included in the analysis, indicating living cells. *p-*values are depicted in the figure. Experiments with a positive result for IgG, IgM, and activated C3 binding after incubation with anti-A2 serum were included in the analysis. Data are normalized to the negative control (blood group- and HLA-compatible serum) and are presented on a logarithmic scale. *p*-values depict results using the unpaired *t*-*t*est. Dots represent individual values.

We crossmatched patient serum with MP-PRECs that were blood group compatible and added rabbit complement as a complement source. Crossmatching with serum taken 1 day prior to transplantation resulted in clear complement-mediated cell death; 72% of all cells appeared to be apoptotic (**Figure 5**). When crossmatching MP-PRECs from 3 different donors with serum taken on POD 3, extensive complement-mediated cell death occurred (*p* = 0.002 compared to blood group- and HLA-compatible serum) (**Figure 6**).

In comparison, control serum from patients with HLA class I directed against HLA antigens expressed on the MP-PRECs caused less complement-mediated cell death (*p* = 0.05) when compared to the patient’s serum (**Figure 6**). When compared to human monoclonal IgG and IgM antibodies directed against HLA-A2, a similar extent of complement-mediated cell death occurred.

Next, we performed custom endothelial flow crossmatch tests with MP-PRECs from 3 different donors, using serum from POD 3. IgM, IgG binding (recognizing human immunoglobulin subclasses IgG1, IgG2, IgG3, and IgG4), and complement activation were analyzed using flow cytometry. To better mirror the physiological situation, 20% human serum was utilized as a complement source. Crossmatching with the patient’s serum resulted in IgG and IgM binding and activated C3 to varying extents (**Figure 6C**). Human monoclonal IgG and IgM antibodies directed against HLA-A2 were used as positive crossmatch test controls, also showing IgG, IgM, and complement binding to varying extents. In addition, detection of non-HLA antibodies in the serum from POD 3 was measured using the multiplex assay developed by the PROCARE Consortium (20), screening for Agrin, APMAP, ARHGDIB, ARHGEF6, AT1R, ETAR, LMNB1, LPLUNC1, PECR PLA2R, PRKCZ, TUBB4B, and vimentin. Only antibodies directed against TUBB4B were found to be present in the patients’ serum (absolute mean fluorescence intensity (MFI) 1,564.5 and control ratio 2.8). However, these antibodies have not been shown to be associated with adverse outcomes after kidney transplantation.

### Second Post-Transplantation Clinical Course

**Figure 7:**
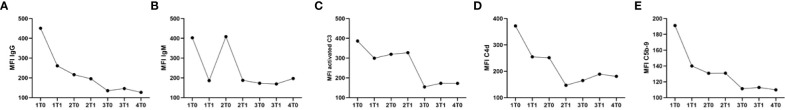
Serum samples collected before and after plasmapheresis show a reduction in the binding of IgG, IgM, activated C3, C4d, and C5b-9 to MP-PRECs during the course of 4 cycles of plasmapheresis. Serum samples were collected right before every plasmapheresis (1, 2, 3, and 4) at the time points (T) before (0) and after (1) every plasmapheresis. **(A)** Binding of IgG to MP-PRECs after incubation with the patient’s serum collected during the course of plasmapheresis. **(B)** Binding of IgM to MP-PRECs after incubation with the patient’s serum collected during the course of plasmapheresis. **(C)** Binding of activated C3 to MP-PRECs after incubation with the patient’s serum collected during the course of plasmapheresis and 20% NHS as a complement source. **(D)** Binding of C4d to MP-PRECs after incubation with the patient’s serum collected during the course of plasmapheresis and 20% NHS as a complement source. **(E)** Binding of C5b-9 to MP-PRECs after incubation with the patients’ serum collected during the course of plasmapheresis and 20% NHS as a complement source.

Two years later, a retransplantation with a renal allograft from a living unrelated altruistic donor was performed. The donor and recipient both tested positive for the Epstein–Barr and cytomegalo viruses, and the donor typing was A1, A2, B7, B60, Bw6, Bw6, Cw10, Cw7, DR13, DR15,DR52, DR51, DQ6, and DQ6 resulting in a HLA A/B/DR mismatch of 1-1-0, again blood group compatible and without DSAs. Transplantation was performed after a desensitization procedure consisting of rituximab (750 mg) 9 days before transplantation and 5 sessions of plasmapheresis with exchange of 1 plasma volume per session and albumin 5% substitution. The plasmapheresis regimen was started 6 days before transplantation, followed by 0.1 g/kg IVIg (Nanogam) and 1 g/kg IVIg after the final session the day before transplantation. The last anti-GBM titer before transplantation was 1 IU/ml. Posttransplantation, plasmapheresis was continued for 3 days followed by 0.1 g/kg IVIg on the first 2 days and 1 g/kg IVIg on the 3rd day. When the patient serum obtained during desensitization treatment was analyzed for the presence of AECA utilizing MP-PRECs, a time-dependent decrease in IgG, IgM, C3, C4d, and C5b-9 was observed (**Figure 7**), suggesting an effective removal of non-HLA AECA. No surgical or other posttransplantation complications occurred (cold ischemia time, 152 min; warm ischemia time, 37 min), and the patient was discharged with an estimated glomerular filtration rate (eGFR) of 44 ml/min/1.73 m^2^ (21). After 2 years, he had a stable allograft function with an eGFR of 41 ml/min/1.73 m^2^.

## Discussion

Although the relevance of non-HLA-related humoral rejection is increasingly recognized in solid organ transplantation, its diagnostic identification and monitoring upon treatment have not been sufficiently defined (22). In this report, we describe the accelerated rejection of a blood type-compatible, living related donor kidney, where HLA-DSA could not be detected pre- or posttransplantation. A biopsy showed features of C4d-ABMR, including extensive hemorrhagic areas and loss of renal vascular endothelial cells. AECAs were identified in crossmatch assays using MP-PRECs from various donors. CDC tests revealed complement-dependent cell death in both pre- and posttransplant serum, most likely representing preformed non-HLA antibodies and not *de novo*. After desensitization for presumed AECA, a successful second transplantation was performed. Using the MP-PREC crossmatch assay, we describe monitoring of the effective reduction of complement-mediated renal EC cytotoxicity upon treatment before the second transplantation. Plasmapheresis to treat non-HLA-related humoral rejection has been described before (23). However, to the best of our knowledge, this is the first report describing meaningful diagnostic monitoring of AECA desensitization strategy in a kidney transplant recipient.

We showed that MP-PRECs can be utilized instead of lymphocytes in the CDC. However, by using rabbit complement in crossmatch assays, we are likely missing the effects of complement regulation that are crucial in the susceptibility to inflammation of endothelial cells (24). Instead, crossmatching using a human complement source showed that the MP-PRECs are heterogeneous in terms of their response to inflammation stimuli.

Histopathological analysis of the patient’s biopsy in **Figures 2**, **4** emphasizes that renal vascular structures might not be equally involved in rejection processes, as endothelial damage and loss were most prominent in peritubular capillaries. MP-PRECs are primary renal ECs originating from different vascular structures in the kidney (peritubular capillaries, glomerular, macro- and microvascular). Therefore, MP-PREC-based CDC is likely representative of an *in vitro* model, as MP-PRECs adequately mirror the immunological situation in its broad vascular heterogeneity and responses to inflammatory stimuli (10). Our assay with MP-PRECs from various donors makes it possible to prospectively screen for AECA and monitor desensitization before transplantation, as we describe for the second kidney transplantation. It is important to mention that Pereira et al. described in 2016 the case of a patient who experienced a hyperacute rejection despite a negative EC crossmatch test, probably due to anti-DQ DSA detected by solid-phase tests based on Luminex technology (25). However, it has been described that renal microvascular endothelium constitutively expresses DR without the other class II proteins DQ and DP (26), thus minimizing the responsibility of anti-DQ antibodies in acute rejection episodes. Nevertheless, it is important to realize that the MP-PREC crossmatch assay might not detect all possible harmful DSAs if the antigen is not expressed on the MP-PREC cell membrane. A role for anti-GBM antibodies as a contributing factor to the renal damage seems highly improbable, as the anti-GBM titers remained low during the clinical course posttransplantation and the histological pattern in the kidney biopsy was not compatible with the anti-GBM disease. Besides, to our knowledge, anti-GBM disease recurrence in a transplant has not been reported to present with fulminant hyperacute rejection as described in this report (27, 28).

The observation of the absence of C4d *in vivo* is similar to the reports about AECA in kidney transplantation described by Jackson et al. and Ronda et al., who found IgG and IgM binding to endothelial progenitor cells and the cell line EA.hy 926 *in vitro* (4, 22). In a later study, Jackson et al. described that patients who were categorized as intermediate or strongly positive for non-HLA Abs measured by ELISA had higher histological scores of microvascular injury, and non-HLA abs identified through a proteomics approach were able to stimulate expression of adhesion molecules and cytokines, albeit *in vitro*. The effect of antibody-mediated damage could also be due to complement-independent mechanisms *via* a mechanism referred to as antibody-dependent cellular cytotoxicity (ADCC). Accumulating evidence suggests that natural killer (NK) cells are important mediators of ADCC in ABMR, and it is likely that multiple parallel mechanisms of endothelial activation contribute to the development of ABMR (29–35).

For example, immune cells like granulocytes could induce injury to the graft endothelium *via* ADCC (36, 37). The kidney biopsy from the patient described here showed infiltration of granulocytes. It has been described that, depending on the FcƴR subclass, effector cells like NK cells, macrophages, monocytes, and granulocytes can contribute to ADCC (38–40). On the other hand, neuthrophils are known to act hand in hand with the complement system to defend the host against invading pathogens. Neuthrophils can produce complement factors themselves, and this may contribute to local inflammation (41). We refer to the recently published review by Lebraud et al. for a detailed discussion on the mechanisms of microvascular damage to the allograft in the absence of HLA antibodies (36).

We acknowledge several limitations of the study. The usefulness of primary endothelial cells could be limited by early onset of senescence and shifts in *in vivo* gene expression due to the loss of microenvironmental cues. In addition, the cell isolation can be time-consuming and expensive, and the success rates might depend on the experience of the researcher. To reproduce this crossmatch assay, it is important to create immortalized cell lines of the primary ECs derived from the machine perfusate, based on their renal origin and antigen expression. However, cell lines also have their limitations that should not be underestimated since the process of immortalizing can cause epigenetic changes that alter their phenotypes. Cell lines could change over time due to chromosomal changes during their growth in the lab after generation (42). Therefore, these cell lines would need a thorough validation that focuses on the persistence of expression of endothelium-specific markers.

In addition, as with all laboratory assays, the crossmatch test presented here needs extensive validation, addressing the sensitivity, specificity, intra/interassay variability, and the assessment of positive thresholds and controls. The case we describe is a proof of principle using cultured endothelial cells from different endothelial donors, showing that in all donor-specific endothelial cells, antibody-mediated complement activation can be seen, although to a different extent. It has to be noted that the validation process might take a reasonable amount of time since this heterogeneity in response to non-HLA antibodies may actually reflect true differences in response mechanisms to non-HLA antibodies between individual donors and may explain some of the variation in outcome in the presence of non-HLA antibodies.

The discovery of new targets for non-HLA antibodies is an ongoing process (4, 19, 43–49). Although several non-HLA antibody specificities have been identified, the currently available solid-phase assays are limited to the known non-HLA antigens (23, 50). Only antibodies directed against TUBB4B were found to be present in the serum of the patient described here; however, this antibody has never been described to correlate with rejection or graft failure (20). It has been described that TUBB4B is ubiquitously expressed in patients undergoing chronic hemodialysis, and it might therefore not be surprising that we detect this antibody in the patients’ serum (19, 51). The pathogenic effect of TUBB4B as the driving force of the hyperacute rejection described here remains questionable, as it seems likely that not all relevant antigens are included in the currently available assays. Differentiation in pathogenic and nonsignificant non-HLA (auto)antibodies remains an important unanswered question to date. The advantage of the cell-based assay is that it could be used as a screening tool, and all pathogenic effects are measured at once. The disadvantage is that the culprit protein remains unknown. Lamarthée et al. recently described the relevance of a tool to identify pathogenic non-HLA antibodies in kidney transplant recipients by utilizing CRISPR/Cas9-engineered HLA-silenced glomerular endothelial cells (50). Their endothelial crossmatch test identifies non-HLA Abs and strongly predicts graft endothelial injury, independent of HLA-DSAs. The authors suggest that HLA-silenced renal endothelial cells from several donors with various genetic backgrounds could provide a wider and more mixed genetic representation, ensuring a more universal cell-based assay. This would be important in order to capture more donor-specific non-HLA Abs, targeting polymorphic proteins. We think that the MP-PREC isolation and crossmatch test described in our report could be an attractive way to go. We recognize that it will be important to compare our assay with other EXCMs, such as the XM-One that uses tie2-positive endothelial progenitor cells (6, 52–54). In addition, we have not determined the specificity of the antibody reacting with the MP-PRECs, yet. This identification of the actual antigen may be useful to identify new relevant non-HLA antigens and develop simpler assays.

We hypothesize that our crossmatch test might add significant value to the characterization of non-HLA antibodies and their detection in terms of standardized diagnostic tests, monitoring therapeutics and for risk stratification prior to transplantation. As organ offers often happen at unexpected moments, a hands-on and quick screening assay for antiendothelial cell antibodies is needed. Also, for HLA-sensitized patients, a panel of multiple endothelial cell donors might be necessary, if the cells are not made devoid of HLA antigen expression. As it is expensive and time-consuming to have endothelial cells in culture without an immediate purpose, a possible suggestion is to grow endothelial cells from multiple donors on cover slips after which the cells could be fixed and stored for future use. We foresee this renal endothelial cell-based crossmatching assay to be instrumental in further unraveling the pathogenicity of antiendothelial cell antibodies.

## Data Availability Statement

The raw data supporting the conclusions of this article will be made available by the authors, without undue reservation.

## Ethics Statement

The study was approved by the University Medical Center Groningen institutional review board (METc 2008/186), adheres to the Declarations of Helsinki and Istanbul and has NCT02811835 as ClinicalTrials.gov identifier. The patients/participants provided their written informed consent to participate in this study.

## Author Contributions

RL, JB, and SB conceived and designed the research. RL, MH-K, AG-N, and AD collected samples. RL and MH-K performed the experiments. RL, MH-K, and AD analyzed data. RL, MD, JB, AD, and SB interpreted the results of the experiments. RL prepared the figures. RL, JB, AD, and SB drafted the manuscript. RL, JB, MD, BH, JS, RP, AD, and SB edited and revised the manuscript. RL, JB, MD, AD and SB approved the final version of the manuscript. All authors listed have made a substantial, direct, and intellectual contribution to the work and approved it for publication.

## Funding

COMBAT Consortium. This study was supported by the Dutch Kidney foundation (grant number: 13O CA27).

## Conflict of Interest

The authors declare that the research was conducted in the absence of any commercial or financial relationships that could be construed as a potential conflict of interest.

## Publisher’s Note

All claims expressed in this article are solely those of the authors and do not necessarily represent those of their affiliated organizations, or those of the publisher, the editors and the reviewers. Any product that may be evaluated in this article, or claim that may be made by its manufacturer, is not guaranteed or endorsed by the publisher.

## Abbreviations

ABMR, antibody-mediated rejection; AECA, antiendothelial cell antibodies; anti-GBM, anti-glomerular basement membrane; CDC, complement-dependent cytotoxicity; CiGeNC, renal glomerular endothelial cell line; DSAs, donor-specific-antibodies; EC, endothelial cells; ECXM, endothelial cell crossmatch; e-GFR, estimated glomerular filtration rate; ERG, ETS-related gene; HLA, human leukocyte antigen; IgG, immunoglobulin G; IgM, immunoglobulin M; IVIg, intravenous immunoglobulin; LSA, Luminex single-antigen bead assay; LSdL, LifeScreen Deluxe; MP-PREC, machine perfusion-derived primary renal endothelial cells; POD, postoperative day.
