# Supplementary material for: Renal Endothelial Cytotoxicity Assay to Diagnose and Monitor Renal Transplant Recipients for Anti-Endothelial Antibodies
Source: Front Immunol. 2022 Jun 6;13:845187. doi: 10.3389/fimmu.2022.845187 (PMC9207246; doi:10.3389/fimmu.2022.845187)
Supplement: Supplementary file 1 [file Table_1.docx]

**Supplementary table 1. Details on the primary antibodies and conjugates used in staining procedures**

| **Marker** | **Antibody** | **Conjugate** |
| --- | --- | --- |
| CD34 | Ventana bench machine, mouse anti-human CD34 (QBEnd/10,Ventana, Mannheim, Germany) |  |
| ETS-related gene (ERG) | Ventana bench machine, rabbit anti-human ERG (ERP3864, Ventana, Mannheim, Germany) |  |
| IgG | Ventana bench machine, goat anti-human IgG (760-2680, Ventana, Mannheim, Germany) |  |
| IgM | Ventana bench machine, goat anti-human IgM (760-2682, Ventana, Mannheim, Germany) |  |
| C4d | Ventana bench machine, rabbit anti-human C4d (SP91, Ventana, Mannheim, Germany) |  |
| C3d | 1:1000 rabbit anti-human C3d, (Dako,  A0063, Glostrup, Denmark) | 1:10000 anti-human C3d-DIG, (Dako) and 1:8000 polyclonal anti-DIG, (Roche, 11207733910) |
| IgG | 20µl APC conjugated mouse anti-human IgG (G18-145, BD biosciences, San Jose, USA) |  |
| IgM | 5 µl PE conjugated mouse anti-human IgM (MHM-88, Biolegend, San Diego, CA, USA) |  |
| Activated C3 | 1:50 Mouse anti-human activated C3 (HM2168, Hycult, Uden, The Netherlands) | 1:100 Goat anti-mouse IgG FITC (Cat. No. 1031-02, SouthernBioTech, Uden, The Netherlands) |
| C4d | 1:100 Mouse anti-human C4d (12D11, Hycult, Uden, The Netherlands) | 1:100 Goat anti-mouse IgG FITC (Cat. No. 1031-02, SouthernBioTech, Uden, The Netherlands) |
| Neoantigen C9 | 1:100 Mouse anti-human neoantigen C9 (HM2264, Hycult, Uden, The Netherlands) | 1:100 Goat anti-mouse IgG FITC (Cat. No. 1031-02, SouthernBioTech, Uden, The Netherlands) |
